# Exploring the Genetic Link Between Coat Colour and Morphological Traits: The Case of Peruano de Paso Horse

**DOI:** 10.3390/ani15182720

**Published:** 2025-09-17

**Authors:** Ayelen Karlau, Florencia Azcona, Antonio Molina, Pablo Trigo, Juan Pablo Sánchez-Serrano, Sebastián Demyda-Peyrás

**Affiliations:** 1 Departamento de Producción Animal, Facultad de Ciencias Veterinarias, Universidad Nacional de La Plata, Calle 60 y 118 s/n, La Plata 1900, Argentina; 2 Consejo Nacional de Investigaciones Científicas y Técnicas (CONICET)-CCT La Plata, Calle 8 1467, La Plata 1900, Argentina; 3 Cátedra de Medicina Equina, Departamento de Clínicas, Facultad de Ciencias Veterinarias, Universidad Nacional de La Plata, Calle 60 y 118 s/n, La Plata 1900, Argentina; 4 Departamento de Genética, Campus de Rabanales, Universidad de Córdoba, CN-IV Km 396, 14014 Córdoba, Spain

**Keywords:** morphology, inbreeding, genetic parameters, heritability, functionality

## Abstract

The Peruano de Paso is a horse breed renowned for its smooth gait, traditionally selected based on breeders’ empirical knowledge. Recently, the frequency of chestnut horses, a recessive trait, has increased substantially within the official studbook. This study evaluated whether preference for chestnut coat colour has influenced morphological traits and genetic diversity within the breed. A total of 8376 records of seven morphological traits, collected by the Peruano de Paso Breeders Association over eight years, were analysed, along with a pedigree including 41,357 individuals. Morphological traits were studied through univariate genetic models that considered potential heterogeneity of additive genetic variance between chestnut and non-chestnut horses. Results indicated moderate to high heritability estimates across traits. No significant differences were observed between groups, except for croup width, with heritability of 0.28 ± 0.08 in chestnuts and 0.44 ± 0.08 in non-chestnuts. Genetic correlations between groups could not be precisely estimated due to limited data. The main effect of selection for chestnut coat colour was a reduction in genetic variability, an increase in inbreeding and relatedness. However, this selection has had little impact on morphology, indicating that the traits valued by breeders remain almost unaffected despite the loss of diversity.

## Simple Summary

The Peruano de Paso (PP) horse is famous for its four-beat, distinctive gait known as “*Paso*”. In this breed, the percentage of chestnut horses (a recessive trait) has increased dramatically in recent decades, fostered by the genetic selection performed. This study aims to determine the genetic influence and relationship of this increase on key morphological traits. To this end, we characterised the PP population and estimated genetic parameters for 7 morphological traits using a phenotypic database of 1047 chestnut and non-chestnut individuals. Estimates for genetic trends indicate that selection towards chestnut coats had a negligible influence on the morphological traits studied. This selection is strongly associated with a reduction in genetic diversity among chestnuts, and some traits show early signs of inbreeding depression. To our knowledge, this is the first study on the genetic basis of morphology in the Peruano de Paso breed, and it suggests only a very weak connection between morphological traits and coat colour.

## 1. Introduction

The Peruano de Paso (PP) is a horse breed globally recognised for its unique, smooth ride and particular gait. Its origins trace back to the 16th century with the introduction of Barb and Andalusian horses by Spanish conquerors [1]. The PP is now the most important horse breed in Peru, officially designated as a National Heritage in 1992. Although the breed originated in Peru, where its main population remains, it is also bred and officially registered in countries such as Ecuador, Argentina, and Panama, with smaller populations in the United States, Colombia, and others. In these regions, dedicated associations support their breeding and management [2]. In Peru, the breed is regulated by the Asociación Nacional de Criadores y Propietarios del Caballo Peruano de Paso (ANCPCPP), which oversees the studbook, registration, and preservation of its legacy [2]. According to their standard, the PP horses are mesomorph, harmonic, and well-proportioned. Individuals should have high withers, a short, straight, broad, and solid dorsum, a robust and wide chest, and a short and moderately sloping croup. In terms of coat colours, they can range from simple to compound coats, including bay, black, and chestnut. On the contrary, overo, tobiano, and albino horses cannot be enrolled according to breed bylaws [2]. However, the most important quality of PP horses is their ability to perform a particular four-beat gait (known as *Paso*). This gait has a predominance of their lateral bipeds, a large extension and flexion in the forelimbs, and a smooth movement in the hind limbs [2]. This gait is performed at high velocities, requiring optimal physical condition and correct morphological conformation. These factors help prevent early injuries that could limit performance, longevity, and overall well-being.

Conformational and morphological traits are central in most horse breeding programmes, significantly influencing mating decisions. Therefore, matings aim to improve the aesthetics of individuals, even more in breeds in which their competitions are primarily morphological [3]. In some others, morphology must be combined with functionality, as competitions include judgments for movements or results for performance [4,5]. In those breeds, mating decisions are also oriented to enhance a horse’s functionality, movement, competitive ability, together with aesthetics [6]. But conformation traits can also serve as early indicators of a young horse’s genetic quality before it is trained for competition [7]. This auxiliary use of morphological traits could be important in some breeds, even determining if a given horse is worth further training. Ultimately, adequate morphological conformation is essential for functionality. This also explains why most formal breeding programmes focus on predicting breeding values for these key traits [8].

Coat colour is an important aesthetic trait in horse conformation and has been used as a selection criterion since domestication [9]. Today, specific coat colours are mandatory in the breed standards of several populations, whereas colour remains a widely applied selection criterion in others [4,10]. Previous studies have also reported associations between coat colour and variation in morphological traits [11,12], suggesting that certain morphological phenotypes may be linked to a particular coat colour. Biologically, this association may be explained by the hypothesis that some genes controlling coat colour are linked to genes by pleiotropy or genetic linkage, thus influencing morphological traits. Interestingly, studbook records show a significant increase in chestnut-coated individuals in the Peruano de Paso breed over the last few decades. Since the chestnut coat results from a recessive allele [13], such an increase is hard to achieve by chance in a population with coat colour variability, such as the PP. This trend raises the question of whether this coat colour could be associated with some morphological or functional changes across the PP population.

Horse breeding programmes are frequently focused on selecting animals with morphological traits that enhance their athletic performance or functionality. Some breeds perform this selection just based on their own phenotypical records, i.e., mass selection, as seen in Criollo Argentino [14] or Polo [15]. In contrast, other breeds select the animals based on estimated breeding values (EBVs) obtained using animal models; examples include Pura Raza Español [16], Murgese [17], and Menorquin [18]. In South America, EBVs are being estimated only in a few breeds, including in Campolina [11] and Criollo Brasileño [8]. The use of EBVs increases the accuracy of the results, reducing the influence of environmental factors on the progeny predictions. This advantage is not yet realised in the Peruano de Paso (PP), where breeding decisions continue to rely on phenotypic values and subjective assessments by breeders. Since the ANCPCPP has taken official records of morphological traits in several tournaments during the last 15 years, the possibility of performing an initial genetic evaluation of the breed is highly valuable.

The aim of this study was to understand the consequences of the increase in chestnut coat colour selection on the population structure of the PP breed; and to estimate genetic parameters and genetic trends of morphological traits of interest, assuming a genetic heterogeneity associated with the horses’ coat colour.

## 2. Materials and Methods

### 2.1. Genealogical and Phenotypical Data

**Figure 1:**
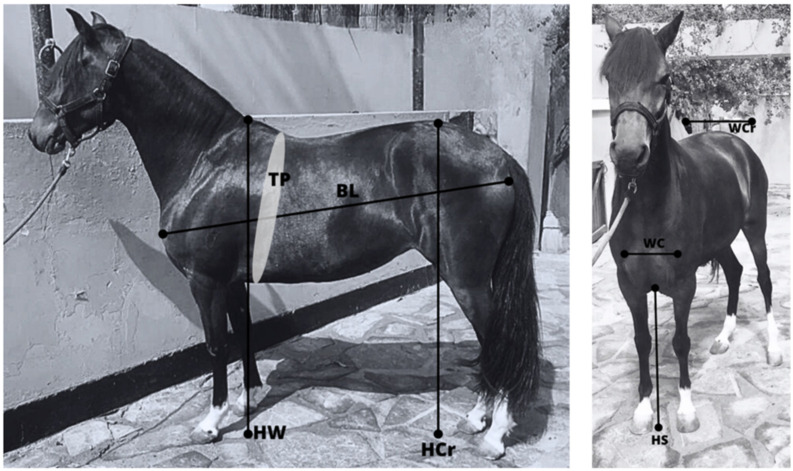
Graphical representation of the morphological measurements taken in the Peruano de Paso horse breed. HW: height at withers, HCr: height at croup, HS: height at sternum, WCr: width of croup, WC: width of chest, BL: body length, and TP: thorax perimeter.

Pedigree records were obtained from the ANCPCPP official studbook, including 41,357 individuals, of which 39,354 had registered coat data. Phenotypic records for seven morphological traits determined by ANCPCPP technicians during official tournaments between 2015 and 2023 were also provided. The traits analysed were height at withers (HW), height at croup (HCr), height at sternum (HS), width of croup (WCr), width of chest (WC), body length (BL), and thorax perimeter (TP) (Table 1 and Figure 1). Following an initial review, extreme and/or erroneous values were removed, resulting in a final dataset of 8376 morphological records from 1047 individuals (862 chestnut and 185 non-chestnut individuals) born between 2001 and 2015. Individuals were grouped based on their coat colour into two groups: chestnut and non-chestnut, to determine the influence of this specific coat colour in the PP breed genetic population and phenotypic structure. The non-chestnut group (17.67% of the morphology population) was comprised primarily of bay (13.56%) and other colours (1.44%). Only 2.67% of the breed was declared as grey. The phenotypic characterisation of the breed was conducted by assessing differences between coat colour and sex. This was performed based on the marginal posterior distributions of the systematic effects from the model used for the genetic analysis; see below.

### 2.2. Population Genetic Characterisation

In a first step PP population was analysed for variability, pedigree robustness, and demographic structure using *optiSel* package [19] from R [20] and ENDOG v4.8 [21]. The evaluation included estimates of the average number of known equivalent (AEG), maximum (AMG), and complete (ACG) generations, inbreeding coefficient (F), and average relatedness (AR). Parameters were assessed at two levels: first, for the whole population, and then separately for two subgroups, chestnuts and non-chestnuts, with morphology records.

### 2.3. Estimation of Genetic Parameters

In a second step, comprehensive genetic evaluations for the seven morphological traits were performed employing a Bayesian MCMC inference methodology. The employed methods allowed the estimation of variance components and genetic parameters, and this was performed using univariate models, whose scalar equation was the following:

$$y_{iklh}=\mu +C_{k}+S_{l}+Y_{h}+\beta_{k}\times F_{i}+a_{i,k}+e_{iklh}$$

where the phenotypic record ($y_{iklh}$) on the *i*th animal, is explained by an overall mean μ; by the effect of the *k*th level (two categories: chestnut and non-chestnut) of coat colour ($C_{k})$; the effect of the *l*th level (two categories: males and females) of sex ($S_{l}$); the effect of the *h*th level (three categories: less than 6 years old; between 6 and 12 years old and older than 12 years old) of age at the time of data recording ($Y_{h}$); the effect of the coat-colour-specific inbreeding depression ($\beta_{k})$, which it was computed as the linear regression on the inbreeding coefficient of each animal with record ($F_{i}$); and finally the effect of an individual coat-colour-specific additive genetic value ($a_{i,k}$); additionally an homoscedastic residual term was considered ($e_{iklh}$).

To better describe the distributional assumptions of the model terms, a matrix representation could be most convenient:

y=Xβ+Za+e

where the vector β includes the above-described systematic effects (coat colour, sex, age and inbreeding depression), and a is a vector of breeding values, containing two realisations per animal in the pedigree, one while being chestnut and another while not being chestnut. X and Z are the corresponding design matrices, for the case of  X, the last column would be the inbreeding coefficient of the animal generating the phenotypic record, and each row of Z would include one, selecting the appropriate breeding value depending on whether the animal is chestnut or non-chestnut.

Under this model specification, the following distributional assumption about the phenotypic records was adopted to accomplish the intended Bayesian study:

$$p(y|\beta ,a,X,Z)\;\sim \;MVN\left(X\beta +Za,R\right)$$

where R is the residual covariance matrix between the observations, this matrix was assumed to be diagonal ($R=I\times \sigma_{e}^{2}$), i.e., records were assumed to be conditionally independent. In a Bayesian analysis, prior assumptions must be adopted for all the model unknowns. In this regard, the terms in β were assumed to follow independent uniform distributions, and the vector of breeding values (a) was assumed to follow the next multivariate normal distribution:

$$p(a|G_{0},A)\;\sim \;MVN\left(0,A⊗G_{0}\right)$$

where A is the additive genetic relationship matrix and $G_{0}$ is a two-by-two symmetric matrix containing the additive genetic variance of the trait in chestnut animals ($\sigma_{a,c}^{2}$), the additive genetic variance of the trait in non-chestnut animals ($\sigma_{a,n}^{2})$ and the additive genetic covariance ${(\sigma }_{a,c-n})$ between both coat colour conditions.

$$G_{0}=\left[\begin{array}{cc} \sigma_{a,c}^{2} & \sigma_{a,c-n}\\ \sigma_{a,c-n} & \sigma_{a,n}^{2} \end{array}\right]$$

In a final hierarchical level, the assumed distributions for the variance components ($\sigma_{e}^{2}$ and $G_{0}$) were uniforms under the valid parameter spaces, i.e., for the variances of the positive real numbers.

These assumptions about the data generation process, as well as the considered prior distribution for the model parameters, yield standard fully conditional distributions [22] that can be sampled using the Gibbs sampler algorithm. For each trait under study, this algorithm was independently run using GIBBSF90+ programme from BLUPF90 family [23]. The Gibbs was run for one million iterations, discarding the first 100,000 as a burn-in period, and afterwards retaining one out of 10 samples for characterising the marginal posterior distribution of the different parameters of interest.

From raw samples of model parameters, i.e., systematic effects, breeding values and variance components, other quantities of interest were computed to generate marginal posterior samples of them. These quantities were heritabilities, genetic correlations and averages of the breeding values of animals born within five-year periods; these last quantities are relevant to assess the genetic trends for the traits under study. In addition to the coat colour-specific heritability, also an overall heritability value was computed applying the following equation to each sample of $G_{0}$.

$$h_{overall}^{2\left[t\right]}=\frac{\left[\begin{array}{cc} n_{c} & n_{n} \end{array}\right]\times G_{0}^{\left[t\right]}\times \left[\begin{array}{c} n_{c}\\ n_{n} \end{array}\right]}{\left[\begin{array}{cc} n_{c} & n_{n} \end{array}\right]\times G_{0}^{\left[t\right]}\times \left[\begin{array}{c} n_{c}\\ n_{n} \end{array}\right]+\sigma_{e}^{2[t]}}$$

where the superscript *t* refers to the *t*th iteration of the Gibbs sampler, and $n_{c}$ and $n_{n}$ are the number of chestnut (862) and non-chestnut (185) animals with phenotypic records.

All this postgibbs analysis was conducted using the POSTGIBBSF90 v2.96 software [24] as well as several R packages (R v4.5) to compute effective sample sizes, Geweke diagnosis statistics, and standard statistics of the marginal posterior distributions. In addition, the *ggplot2* v3.5.2 [25] R package was used for the graphical representation of the different parameters of interest.

## 3. Results

### 3.1. Demographic Structure of the Breed

The initial demographic characterisation of the breed showed a robust pedigree structure, averaging 12.32 ± 4.29 maximum (AMG), 3.84 ± 1.31 complete (ACG), and 6.11 ± 1.81 equivalent (AEG) generations, among individuals with known pedigree. In terms of census, the breed demonstrated strong vitality, with an average of 948 new foals enrolled annually during the period of morphological data collection (2015–2023). However, the breed exhibited reduced genetic diversity, as indicated by several metrics: the effective number of founders (*Fe*) was 17; the effective number of ancestors (*Fa*) was 11, and only 4 ancestors explained 50% of the genetic variability. This situation is also supported by the trend observed in the average inbreeding coefficient (F) among individuals enrolled each year, which increased from 0.72% in 1970 to 9.67% in 2020.

A similar pattern was detected in both chestnut and non-chestnut groups when analysed separately (Table 2). However, chestnuts showed lower *Fe* (16 vs. 23) and *Fa* (10 vs. 13) values than non-chestnuts, despite both groups having well-established pedigree frameworks. The same differences were observed in F (9.51 ± 4.55 vs. 7.09 ± 4.14) and AR (19.60 ± 4.16 vs. 14.80 ± 3.83) values for chestnuts and non-chestnuts, respectively. All these values indicate a stronger selection pressure and reduced genetic variability in chestnut animals, particularly given their larger population size analysed in this study (862 chestnuts vs. 185 non-chestnuts).

**Figure 2:**
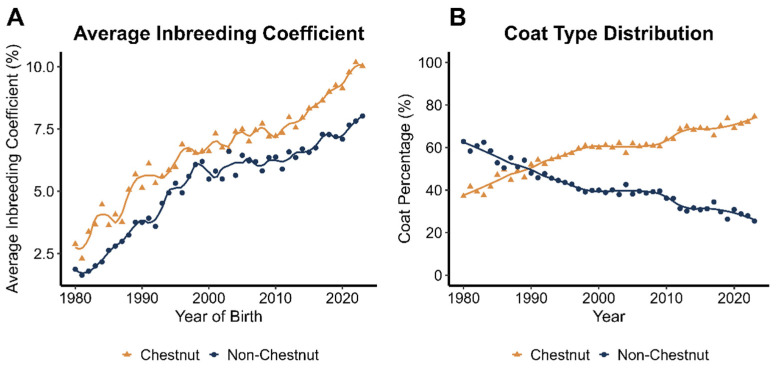
Comparative trends of incidence and inbreeding average value between chestnut and non-chestnut individuals in the Peruano de Paso breed during the last 50 years. (**A**) Average inbreeding coefficient; (**B**) coat colour distribution. Both trends were greater in chestnut.

The incidence and variation in inbreeding between chestnut and non-chestnut individuals over 50 years (1970–2020) are shown in Figure 2. Inbreeding values (F) increased steadily in both subpopulations but were consistently higher in chestnuts (Figure 2A). However, the proportion of chestnut individuals in the pedigree rose notably during the same period, from 25 to 30% of enrolled horses per year in the 1970s to nearly 75% by 2020 (Figure 2B). This increase was particularly pronounced over the last two decades, during which the proportion of chestnuts in the total population grew by an average of 1.2% per year.

### 3.2. Phenotypic Characterisation of Chestnut and Non-Chestnut Horses

The phenotypic characterisation of PP horses is presented in Table 3. On average, the population exhibited a mesomorphic, medium-sized morphology with a large thoracic perimeter. Sexual dimorphism was observed for all the traits under study, in four cases (HW, HCr, HS and WC) the males was larger than the females, while for the other three traits the females had larger phenotype (WCr, BL and TP), despite this statistical result the magnitudes of the differences are rather low, reaching statistical significance due to the reduced variability of the recorded traits (Table 1). Regarding the contrasts between coat colours, no statistically relevant differences were observed for any trait; the contrasts never reached a value higher than one centimetre.

### 3.3. Modelling the Genetic Influence on Morphological Traits

**Figure 3:**
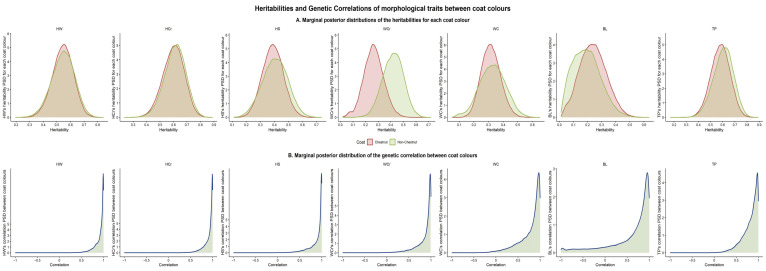
Genetic parameters of morphological traits between coat colours. (**A**) Marginal posterior distribution of the heritabilities of morphological traits; (**B**) marginal posterior distribution of the genetic correlations of morphological traits between coat colours. PSD: posterior standard deviation; HW: height at withers; HCr: height at croup; HS: height at sternum; WCr: width of croup; WC: width of chest; BL: body length; TP: thorax perimeter.

Heritability estimates for the morphological traits are shown in Table 4 and Figure 3A. Overall, h^2^ estimates were moderate to high for most traits, with posterior standard deviations (PSD) up to ten times lower, supporting the robustness of the estimates and a strong genetic contribution to morphological variation. Values were similar between chestnut and non-chestnut horses, ranging from moderate (0.26 ± 0.10 for BL) to high (0.61 ± 0.08 for HCr) in chestnuts, and from 0.21 ± 0.09 (BL) to 0.62 ± 0.08 (HCr) in non-chestnuts. This similarity may suggest no major genetic heterogeneity in the determinism of morphological traits linked to coat colour in this PP population. The only exception was WCr, which showed a slightly divergent heritability between groups (0.28 in chestnut vs. 0.44 in non-chestnut), probably indicating differences in the underlying genetic determinism of this trait across coat colour groups.

The genetic correlations between coat colours for each morphological trait are shown in Table 5 and Figure 3B. Correlations were high, ranging from 0.70 (BL) to 0.93 (HW and HCr), although the associated PSD values were relatively large. Despite these large errors and the asymmetric distribution of the marginal posterior distributions of the genetic correlations (Figure 3B), the overall large point estimates of the correlations align with the lack of genetic heterogeneity across coat colour. Even for WCr, where heritability estimates differed slightly between groups, the strong genetic correlation (0.89 ± 0.17) indicates no genetic heterogeneity between coat colour groups for this trait.

**Figure 4:**
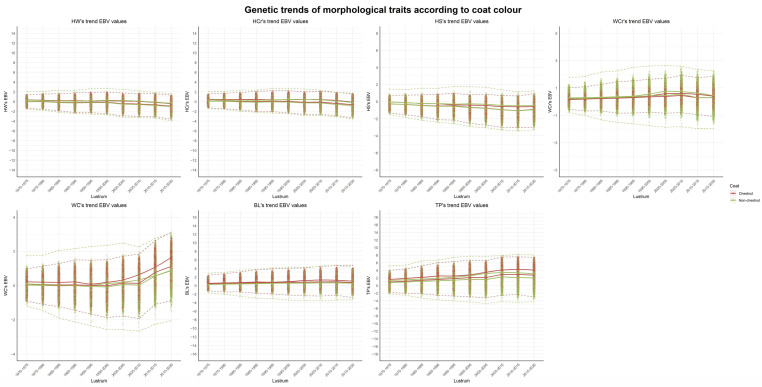
Estimated breeding values for morphological traits between coat colours across lustrums. EBV: estimated breeding values; HW: height at withers; HCr: height at croup; HS: height at sternum; WCr: width of croup; WC: width of chest; BL: body length; TP: thorax perimeter.

Estimated genetic trends, based on averages of five-years period estimated breeding values (EBVs), are shown in Figure 4. The only traits clearly showing significant trends over the last three lustra are WC and WCr. In agreement with the parameter estimates, no relevant difference in the observed trends can be assessed between chestnut and non-chestnut animals. This, again, is evidence to support the rejection of any genetic heterogeneity in the genetic control of the morphological traits between chestnut and non-chestnut colour animals.

Finally, inbreeding depression coefficients were predominantly negative across morphological traits and both coat colour groups, although their magnitude and significance were highly variable (Table 6). The strongest effects in magnitude were observed for thorax perimeter in both groups, but only the estimate in chestnut individuals reached statistical support (*p* > 0 = 0.02). Most other traits also showed negative values, though without significance (*p* > 0 > 0.40). Positive estimates were only observed in height at the sternum but were also non-significant. Notably, the probability values (*p* > 0) were highly inconsistent: while some traits showed virtually no evidence of inbreeding depression (e.g., height at sternum in both groups, *p* > 0 = 0.87 and 0.53, respectively), others displayed moderate to strong support, such as thorax perimeter in chestnut animals (*p* > 0 = 0.02). This suggests that inbreeding does not uniformly affect morphological traits, with some traits being largely unaffected and others showing incipient signs of inbreeding depression. Nevertheless, although current effects are small, monitoring is essential for future breed management.

## 4. Discussion

The Peruano de Paso horse is a breed recognised worldwide for its distinctive gait. Its original genetic nucleus and the most important breeders’ association are in Peru, where these horses have been bred since the late 1950s. Despite their popularity in Peru and across the Americas, the breed’s genetic structure has remained largely understudied to date.

Our results indicate that PP has a robust pedigree, as reflected by an AEG greater than 6. Pedigree reliability is large compared to open enrolment breeds, such as the Brazilian Sport Horse [26] or Spanish Anglo-Arabian [27], whose AMG, AEG, and ACG values were lower. This is an expected finding since not-enrolled individuals can be used as sires or dams under certain circumstances in such breeds, a practice commonly banned in PP. However, the pedigree records analysed showed a higher degree of robustness in comparison with several American closed-enrolment breeds, such as Quarter Horse [28], Criollo Argentino [14] and Brazilian Campolina [29]. In contrast, European breeds with a large history of record availability, such as the Pura Raza Español [30], showed much increased values. Interestingly, the high AMG value reflects the long-standing breed registry, with records preserved for foundation animals and certain “elite” lines whose pedigrees are fully traceable many generations back. In contrast, ACG values were lower than expected for a closed enrolment breed. This discrepancy is likely a consequence of the introduction of unregistered individuals, temporarily allowed a couple of decades ago, as part of an effort to increase the breed’s genetic variability. Despite this period of studbook openness, the pedigree analysis supports the reliability of the results obtained in the present study.

*Genetic variability is reducing, and inbreeding is increasing in the Peruano de Paso breed, particularly in chestnut horses*

The increase in inbreeding, together with a reduction in effective population size, highlights a clear decline in the Peruano de Paso’s genetic variability. Additionally, the small number of ancestors and individuals explaining half of the genetic variation indicates that this reduction has affected both chestnut and non-chestnut horses over recent decades. This pattern could be explained by the lack of limits on the number of foals that a given sire or mare can register per year. The effect is further reinforced by the unrestricted use of artificial insemination (A.I.) and embryo transfer (E.T.) allowed in the breed, with the latter currently accounting for ~20% of the foals enrolled annually. Together, these two factors have indirectly reduced the number of mares and sires that have contributed to the breed, particularly over the past twenty years, when 21,824 foals were registered, resulting from 2161 sires and 6994 dams. The current mating strategies in the Peruano de Paso breed appear to be largely based on empirical knowledge and personal preference of breeders, without a centralised, coordinated selection programme. This has led to the intensive use of a small number of champion stallions, a practice greatly facilitated by the unrestricted use of assisted reproductive technologies. For example, a single sire accounted for approximately 12% of the total registrations in the breed during the last two years analysed, while just seven sires were responsible for around 40% of the total registrations over the same period. The unrestricted combination of A.I. and E.T., was previously associated with decreases in genetic variability and an increase in inbreeding in horses, even in open-enrolment breeds [15]. In contrast, such a reduction in genetic variability was not observed in closely related breeds, such as the Criollo Argentino, where the number of foals per sire and the use of assisted reproductive techniques (ART) are limited by studbook bylaws [14]. Similarly, breeds in which ARTs are employed less intensively do not show this pattern. On the contrary, the ANCPCPP studbook had recently approved the use of cloned horses as breeding animals. This situation could likely increase the loss of genetic variability in the breed by further reducing the number of sires and dams used as breeders and increasing the genetic influence of the cloned founders in the whole pedigree.

It is well known that inbreeding increase is associated with phenotypic problems in many species. In horses, refs. [31] (among others) recently demonstrated that inbreeding negatively affects horse morphology in breeds with open and closed enrolment policies. Our results showed that average inbreeding values per year have more than doubled over the last decades, together with preliminary evidence of inbreeding depression in the morphological traits analysed. The same genetic trend was also described by [1] in Peruvian horses raised outside Peru, suggesting that similar breeding practices are being applied across PP breeders’ associations in Central America and the United States. The recent limitations imposed by the ANCPCPP on the number of foals per sire per year, along with preliminary restrictions on the use of embryo transfer, represent important steps toward acknowledging the issue and mitigating further loss of genetic variability.

*Genetic determinism of morphological traits in the Peruano de Paso horse*

Conformation traits play an important role in horse breeding, influencing results in morphological competitions [32]. However, these traits form the foundation for correct functionality, longevity and performance in breeds in which competencies are based on performance (such as polo or trotters) [33]. Because all conformation traits have a polygenic, quantitative genetic basis, implementing a systematic, population-based approach to genetic evaluation is essential for reducing uncertainty and improving the efficiency of mating outcomes. Here, we modelled for the first time the genetic influence on morphological traits of interest in the Peruano de Paso breed, while also assessing the potential genetic heterogeneity across coat colours.

In general, heritabilities (h^2^) were lower than those reported in other large populations but were higher than those described for small local breeds. For example, we estimated a h^2^ of 0.56 for HW, a trait with relatively high genetic determination. The estimate is lower than the reported for Pura Raza Español (0.80 [7]), a breeding programme with more than 150,000 phenotyped horses, but comparable to estimates for the Campolina breed (0.43 [11]) and Old Kladrub horses (0.44 [34]), both of which were obtained from substantially smaller datasets. Interestingly, the same pattern was observed in the rest of the morphological traits analysed. It is well established that large phenotypic datasets with extensive pedigree information tend to provide more precise estimates and can capture a greater proportion of the additive genetic variance [35]. Nevertheless, our estimates were higher than those reported for smaller, local breeds such as Campolina and Old Kladrub horses, even though they were obtained using a larger dataset. A likely explanation for this increased accuracy is that phenotypic records in the Peruano de Paso are collected exclusively by the breeders’ association, which enhances measurement consistency and reliability, thereby improving the precision of the estimates.

*Peruano de Paso breed is being selected toward chestnut coats without affecting phenotypes*

The diversity of coat phenotypes observed in modern horses has been linked to domestication processes and selective breeding over time [9]. Evidence suggests that selection by coat colour is an ancient practice, established from the beginning of horse breeding by humans. Today, coat colour is a notable characteristic in several breeds: in some, such as Appaloosa [36] and Menorquín [37], it represents a definitive feature and a hallmark for the breed, while in others, specific coat colours are strictly regulated [12]. Importantly, selection for coat colour exceeds aesthetics since it can have broader breeding implications. It may inadvertently shape genetic diversity or influence the frequency of traits linked to those coats, as demonstrated in Appaloosa horse breed [38]. Similar effects have also been reported in other mammal species [39], where selection for coat colour was linked with a reduction in heterozygosity and allelic richness in the whole population.

Longitudinal changes in the frequency of a particular coat colour within breeding populations under artificial selection are generally driven by two factors: breeders’ preferences or functional advantages [40]. In PP, our data revealed an unexpected increase in the incidence of chestnut coats, a recessive phenotype, over the last decades. Even so, no phenotypic differences were detected for the conformational traits evaluated across coats. However, phenotypic differences were detected for the traits of interest between males and females. This was expected due to the presence of sexual dimorphism, which has already been described in other equine breeds [7].

Taken together, these findings suggest that PP selection towards this coat is most likely attributable to breeders’ aesthetic preferences rather than functional advantages. However, the PP breed is also heavily selected for *el Paso*, a functional trait that was not evaluated in this study. Therefore, potential associations between coat colour and functional performance traits cannot be ruled out. In addition, market dynamics may also influence the prevalence of certain coat colours within a breed. For example, in Pura Raza Español, the frequency of Cream [41] and Pearl [42] dilutions has increased, largely driven by the higher economic value attributed to carrier stallions and mares. By contrast, this trend is not evident in PP, where individuals with superior performance in functional traits—particularly *el Paso*—consistently reach the highest market values regardless of coat colour.

It is important to note that some horses classified as non-chestnut were grey. Grey is a dominant coat colour (except in the presence of dominant white) and progressively masks the underlying pigmentation, regardless of whether the horse carries bay or chestnut genotypes [43]. As a result, some horses carrying the chestnut genotype (ee) may have been incorrectly assigned to the non-chestnut group, introducing a potential source of bias. However, only 2.67% of the horses in this study were grey (15.13% of the non-chestnut group), and just 30% of them (0.8% of the total) descended from a mating with at least one chestnut ancestor. Even under this worst-case scenario, fewer than 1% of the total horses analysed could have been misclassified. Given this minimal proportion and the robustness of the overall results, the potential impact of such misassignments on the study’s findings is negligible.

On the other hand, the demographic patterns observed suggest that the intensive use of chestnut sires, likely driven by aesthetic preferences or additional unknown causes, has contributed to an increased frequency of the recessive allele *e* associated with chestnut coats. Our results support this hypothesis, as the chestnut subpopulation exhibited higher average relatedness and inbreeding coefficients and reduced *Fe* and *Fa* compared to non-chestnut individuals. These differences indicate that the selective use of chestnut sires could have influenced the genetic structure of the breed, reducing the genetic variability by triggering a bottleneck effect.

Coat colours have been associated with genetic variation in morpho-functional traits, including behaviour [44,45] and morphology [37,46,47], in several breeds. In contrast, our results indicate that coat colour exerts little influence on the genetic determinism of morphological traits in the PP breed. Six of the seven traits analysed showed no significant differences in heritability between coat-colour groups, and genetic correlations between traits in chestnut and non-chestnut animals were consistently high. This finding is further supported by the genetic trends observed across lustra, which displayed similar patterns in both groups. Even for WCr, which exhibited numerically different heritability estimates between groups, the genetic longitudinal trend pattern was almost identical between groups. However, it is worth mentioning that differences in heritability between chestnut and non-chestnut animals cannot be declared to be statistically relevant, since the uncertainty regions of both heritability estimates largely overlap (Figure 3A). Nonetheless, these differences in the additive effect between coats detected in WCr may suggest a partial linkage disequilibrium between coat colour *loci* and genes involved in the genetic control of the trait. This possible association between coat colour and morphological traits has been previously reported in other species, suggesting that coat colour genes may influence additional phenotypes beyond pigmentation. But such reports pinpoint a pleiotropic effect of the genes as the primarily cause of the association, rather than LD association. For instance, Sanchez-Guerrero, Negro-Rama, Demyda-Peyras, Sole-Berga, Azor-Ortiz and Valera-Cordoba [47] described a subtle pleiotropic effect of coat-colour genes on certain morphological traits, and Bellone [48] associated coat colour with diseases in several horse breeds. Our results indicate that, although coat colour does not explain variation in most morphological traits in this population, there may be a trait-specific association with WCr. But the potential mechanism involved in explaining this association is not clear.

Some studies suggest that including coat colour as a fixed effect in the statistical model may improve the robustness of genetic parameter estimates. For instance, refs. [11] and [49] reported increased accuracy in Campolina and Italian native breeds. However, in our study, no meaningful differences were detected in most traits between coat-colour groups, which suggests that variance heterogeneity across groups is limited. This aligns with findings in other breeds—such as Criollo, Mangalarga [50], Quarter Horses [51], and Thoroughbreds [52]—where coat colour is not included in the models without compromising the reliability of the results. Taken together, these observations indicate that in PP horses, accounting for coat colour in genetic evaluations is unlikely to yield substantial improvements in parameter estimation.

Finally, differences in the influence of coat colour on quantitative traits among breeds have also been associated with population constraints, such as founder or bottleneck effects, or when using highly preselected groups of animals. An example is the Old Warmblood breed, where the population is more uniform because all animals are intended for sport purposes [53]. According to our results, a similar scenario may occur in PP, as phenotypes are mostly collected during competitions, where reduced phenotypic variability is expected due to the intense selection pressure applied to these animals. To better assess this constraint, future studies should incorporate phenotypic data from a broader spectrum of the population, including non-competitive animals, to capture the full range of variability and provide more accurate estimates of genetic parameters.

*Morphological biotype as a selection criterion of interest*

Morphological conformation is a major selection criterion in almost all horse breeds, not only because it affects the animal’s aesthetics and breeders’ preferences, but also because it can enhance performance by favouring individuals with functional body structures [7,31,54,55]. However, most breeding programmes still rely primarily on phenotypic data and pedigree evaluation rather than the use of estimated breeding values (EBVs) to guide mating design and decision-making. This is the case of PP, the current mating strategies appear to be largely based on empirical knowledge and personal preference of breeders, without a centralised, coordinated selection programme [2]. This phenotypical selection has produced great improvements in the quality of some South American breeds, such as Argentine Polo [15], Criollo Argentino [14] and several Brazilian breeds [56]. But the success of this methodology has been particularly driven by selecting traits with high heritability, such as gait-related [57] and conformational traits, for which the own phenotype value represents a moderately accurate predictor of the breeding value.

In PP, breeders often claim that modern horses are “genetically better” than earlier generations, although opinions on this matter remain inconsistent within the community. From a genetic point of view, such perceptions are likely driven by an increased selection intensity, historical bottlenecks, and changes in breeding goals, all of which may have shaped the current genetic architecture of the breed. Using the extensive phenotypic records collected by the breeders’ association, this study is the first attempt to quantify the genetic contribution to morphological traits in PP and to estimate EBVs. This approach has already transformed breeding practices on several breeds and activities, including dressage [58], showjumping [59], mixed abilities [57], fertility [60] and morphology [55]. To our knowledge, only a pilot study was performed estimating heritability in PP. But it only included three functional traits, a small pedigree and a very reduced number of individuals with phenotypes [61]. Therefore, results should be taken with caution.

Estimating breeding values may appear straightforward from a quantitative perspective, it represents a significant cultural and technical step, moving from subjective evaluations based on aesthetics or rider preference toward objective selection tools. Since this methodology is not yet widely implemented in horse breeding, and is even less common in South American breeds, this study could establish the PP as a model for regional breeding programmes. The reliability of our results, together with an adequate model convergence level as well as the high consistency of the results, suggests that the existing dataset is reliable and can be used as a first step in the implementation of a breeding programme in PP horses. Moreover, it opens the path for future integration with modern genomic methodologies (gEBVs), which have recently begun to be developed in horses and have already proven to be more precise and reliable in some breeding programmes [62,63].

*Initial Signals of Inbreeding Depression in PP*

Inbred mating has long been used to fix and homogenise morphological characteristics of interest in horses [29]. However, such practices can trigger inbreeding depression, a genetic phenomenon in which increasing inbreeding negatively affects the average values of phenotypic traits [64]. These unfavourable effects of the inbreeding have been seen in other closed studbook breeds such as Pura Raza Española, Sardinian Anglo-Arab, and Thoroughbreds [31,65,66]. In PP, we detected a substantial increase in inbreeding over the past four decades, which may be a consequence of practising intended inbred mating or just the overuse of certain highly popular stallions. This increase in inbreeding also produced a subtle inbreeding depression in most morphological traits, regardless of coat colour. However, we also detected considerable variability in the estimates for inbreeding effect within the traits, suggesting that some individuals are more strongly affected than others by this genetic constraint. Since all estimations showed large estimation errors, the proper statistical declaration of the negative sign of the depression was obtained only in TP for chestnuts. Therefore, the association between the increase in inbreeding and a reduction in the morphological phenotypes in PP should be taken with caution. Nevertheless, these findings, together with the increase in inbreeding observed in the breed over the last two decades, emphasise the importance of continued monitoring and management. Potential strategies that have been suggested in other breeds include controlling the use of highly popular stallions, maintaining genetic diversity through the selection of less-related individuals, and applying genomic tools to monitor inbreeding and support informed breeding decisions while preserving morphological and functional traits. Some of these approaches are currently under consideration by the ANPCCPP.

## 5. Conclusions

Here, we estimated for the first time the genetic component of morphology traits in the Peruano de Paso breed using a standardised phenotypic dataset. Our results revealed medium to high heritability values for the traits evaluated, indicating good potential for response to selection. We also found that coat colour does not significantly affect breed morphology, suggesting that other factors—such as the breeder preferences or functional advantages—may explain the observed shift toward chestnut individuals in recent decades. Population analyses further revealed a reduction in genetic variability and an increase in inbreeding, providing data supporting the need for an inbreeding monitoring programme in the near future. Overall, our findings provide a foundation for the establishment and development of a structured genetic breeding programme for the Peruano de Paso horse.

## Figures and Tables

**Table 1:** Summary description of morphological measurements.

Trait	Definition	$\bar{x}$ ± SD			
		Chestnut	Non-Chestnut	Males	Females
Height at withers (HW)	Perpendicular distance from the withers that descend vertically to the ground profile.	146.20 ± 3.00	146.90 ± 2.92	147.20 ± 2.92	146.00 ± 2.97
Height at croup (HCr)	Perpendicular distance from the point of highest elevation of the sacroiliac angle to the ground profile.	145.50 ± 2.83	145.80 ± 2.84	145.80 ± 2.84	145.40 ± 2.83
Height at sternum (HS)	Perpendicular distance from the middle third of the sternum (at the level of the olecranon) to the ground.	74.90 ± 2.66	75.46 ± 2.64	76.35 ± 2.15	74.44 ± 2.52
Width of croup (WCr)	Distance between the most lateral points of the iliac crests.	47.77 ± 2.20	47.78 ± 2.39	46.61 ± 2.13	48.25 ± 2.10
Width of chest (WC)	Distance between the most cranial and lateral points of the scapulohumeral point.	33.71 ± 2.80	33.81 ± 2.79	34.54 ± 2.76	33.39 ± 2.75
Body length (BL)	Line from the most cranial and lateral point on the scapulohumeral joint to the extreme angle of the ischium.	153.10 ± 5.06	153.30 ± 4.74	151.90 ± 5.07	153.60 ± 4.90
Thorax perimeter (TP)	Line that goes around the thorax at the level of the spinous process of the seventh to eighth dorsal vertebrae and the lower sternal region.	180.80 ± 6.03	180.80 ± 6.14	178.60 ± 5.58	181.70 ± 6.01

$\bar{x}$: mean; SD: standard deviation. All the measurements in the manuscript are expressed in centimetres.

**Table 2:** Demographic structure of chestnut and non-chestnut subpopulations.

Parameter	Chestnut			Non-Chestnut		
	$\bar{x}$ ± SD	Min	Max	$\bar{x}$ ± SD	Min	Max
AMG	15.67 ± 1.90	8	21	14.91 ± 2.71	3	19
ACG	4.74 ± 0.76	2	7	4.46 ± 0.99	1	6
AEG	7.50 ± 0.70	4	9.52	7.20 ± 1.10	2	8.97
AR	19.60 ± 4.16	5.77	28.81	14.80 ± 3.83	0.59	23.05
F	9.51 ± 4.55	0	32.05	7.09 ± 4.14	0	30.77

AMG: average maximum generations; ACG: average complete generations; AEG: average equivalent generations; AR: average relatedness coefficient; F: inbreeding coefficient; $\bar{x}$: mean; SD: standard deviation; Min: minimum; Max: maximum.

**Table 3:** Marginal posterior means and posterior standard deviations of the least squares means of the phenotypic traits, and of the contrasts between chestnut and non-chestnut animals, and between males and females.

Trait	LSM	Contrast Between Coat Colour Classes (Chestnut–Non-Chestnut)		Contrast Between Sex Classes (Males–Females)	
	PM ± PSD	PM ± PSD	*p* > 0	PM ± PSD	*p* > 0
HW	146.85 ± 0.72	−0.50 ± 0.59	0.19	1.19 ± 0.19	1.00
HCr	145.55 ± 0.71	−0.27 ± 0.58	0.32	0.48 ± 0.18	1.00
HS	76.09 ± 0.59	−0.49 ± 0.51	0.16	1.87 ± 0.17	1.00
WCr	46.72 ± 0.49	−0.21 ± 0.46	0.32	−1.62 ± 0.14	0.00
WC	32.89 ± 0.59	−0.36 ± 0.57	0.26	1.12 ± 0.18	1.00
BL	151.02 ± 1.03	−0.63 ± 1.06	0.27	−1.73 ± 0.34	0.00
TP	176.57 ± 1.46	−0.98 ± 1.34	0.23	−2.97 ± 0.38	0.00

LSM: Least Squares Means; PM: posterior mean; PSD: posterior standard deviation; *p* > 0: probability of the contrast being greater than zero; HW: height at withers; HCr: height at croup; HS: height at sternum; WCr: width of croup; WC: width of chest; BL: body length; TP: thorax perimeter.

**Table 4:** Estimates heritabilities for chestnut and non-chestnut PP horses.

Trait	Overall	Chestnut	Non-Chestnut
	PM ± PSD	PM ± PSD	PM ± PSD
HW	0.56 ± 0.07	0.56 ± 0.08	0.56 ± 0.08
HCr	0.61 ± 0.08	0.61 ± 0.08	0.62 ± 0.08
HS	0.40 ± 0.07	0.41 ± 0.08	0.42 ± 0.09
WCr	0.30 ± 0.08	0.28 ± 0.08	0.44 ± 0.08
WC	0.32 ± 0.06	0.33 ± 0.07	0.34 ± 0.09
BL	0.23 ± 0.09	0.26 ± 0.10	0.21 ± 0.09
TP	0.58 ± 0.08	0.59 ± 0.08	0.61 ± 0.08

PM: posterior mean; PSD: posterior standard deviation; HW: height at withers; HCr: height at croup; HS: height at sternum; WCr: width of croup; WC: width of chest; BL: body length; TP: thorax perimeter.

**Table 5:** Genetic correlations between coat colours.

Trait	PM	PSD
HW	0.93	0.10
HCr	0.93	0.09
HS	0.90	0.15
WCr	0.89	0.17
WC	0.82	0.23
BL	0.70	0.41
TP	0.86	0.15

PM: posterior mean; PSD: posterior standard deviation; HW: height at withers; HCr: height at croup; HS: height at sternum; WCr: width of croup; WC: width of chest; BL: body length; TP: thorax perimeter.

**Table 6:** Inbreeding depression coefficients of morphological traits.

Trait	Chestnut		Non-Chestnut	
	PM ± PSD	*p* > 0	PM ± PSD	*p* > 0
HW	−2.56 ± 2.75	0.18	−1.24 ± 6.04	0.43
HCr	−4.26 ± 2.67	0.06	−4.08 ± 5.93	0.25
HS	2.65 ± 2.33	0.87	0.32 ± 5.24	0.53
WCr	−1.29 ± 1.86	0.25	−6.76 ± 4.79	0.08
WC	−0.94 ± 2.50	0.35	−1.24 ± 5.62	0.42
BL	−1.33 ± 4.64	0.38	−2.02 ± 9.66	0.42
TP	−10.83 ± 5.52	0.02	−16.91 ± 13.09	0.10

PM: posterior mean; PSD: posterior standard deviation; *p* > 0: probability of the coefficient being greater than zero; HW: height at withers; HCr: height at croup; HS: height at sternum; WCr: width of croup; WC: width of chest; BL: body length; TP: thorax perimeter.

## Data Availability

Restrictions apply to the availability of these data. Pedigree and morphological records are the property of Asociación Nacional de Criadores y Propietarios de Caballos Peruanos de Paso (ANCPCPP) and were provided for this study under a non-commercial confidentiality agreement. Data is available from ANCPCPP upon request and with their permission.

## Abbreviations

The following abbreviations are used in this manuscript:

PP	Peruano de Paso
ANCPCPP	Asociación Nacional de Criadores y Propietarios del Caballo Peruano de Paso
EBVs	Estimated Breeding Values
HW	Height at withers
HCr	Height at croup
HS	Height at sternum
WCr	Width of croup
WC	Width of chest
BL	Body length
TP	Thorax perimeter
AEG	Average number of known equivalent generations
AMG	Average number of known maximum generations
ACG	Average number of known complete generations
F	Inbreeding coefficient
AR	Average relatedness
MCMC	Markov chain Monte Carlo
*Fe*	Effective number of founders
*Fa*	Effective number of ancestors
LSM	Least Squares Means
PM	Posterior mean
PSD	Posterior standard deviations
*p* > 0	Marginal Posterior Probability of the contrast being larger than zero
A.I.	Artificial insemination
E.T.	Embryo transfer
ART	Assisted reproductive techniques
LD	Linkage disequilibrium
